# Molecular Interactions of Antibody Drugs Targeting PD-1, PD-L1, and CTLA-4 in Immuno-Oncology

**DOI:** 10.3390/molecules24061190

**Published:** 2019-03-26

**Authors:** Hyun Tae Lee, Sang Hyung Lee, Yong-Seok Heo

**Affiliations:** Department of Chemistry, Konkuk University, 120 Neungdong-ro, Gwangjin-gu, Seoul 05029, Korea; hst2649@naver.com (H.T.L.); dltkdgud92@naver.com (S.H.L.)

**Keywords:** crystal structure, immune checkpoint, PD-1, PD-L1, CTLA-4, cancer, therapeutic antibody

## Abstract

Cancer cells can evade immune surveillance through the molecular interactions of immune checkpoint proteins, including programmed death 1 (PD-1), PD-L1, and cytotoxic T lymphocyte-associated antigen 4 (CTLA-4). Since 2011, the FDA-approved antibody drugs ipilimumab (Yervoy^®^), nivolumab (Opdivo^®^), pembrolizumab (Keytruda^®^), cemiplimab (Libtayo^®^), atezolizumab (Tecentriq^®^), durvalumab (Imfinzi^®^), and avelumab (Bavencio^®^), which block the immune checkpoint proteins, have brought about a significant breakthrough in the treatment of a wide range of cancers, as they can induce durable therapeutic responses. In recent years, crystal structures of the antibodies against PD-1, PD-L1, and CTLA-4 have been reported. In this review, we describe the latest structural studies of these monoclonal antibodies and their interactions with the immune checkpoint proteins. A comprehensive analysis of the interactions of these immune checkpoint blockers can provide a better understanding of their therapeutic mechanisms of action. The accumulation of these structural studies would provide a basis that is essential for the rational design of next-generation therapies in immuno-oncology.

## 1. Introduction

Immune responses mediated by T cells are tightly regulated by costimulatory and coinhibitory mechanisms, providing an optimal balance between immune reactivity to antigens and maintenance of self-tolerance [1,2,3]. Immune checkpoints play key roles in negative-regulatory pathways of the immune system that function to maintain self-tolerance by protecting the host from autoimmunity [4,5,6,7,8,9]. Cancer cells express tumor-specific antigens derived from genetic and epigenetic alterations, which causes T-cell immune responses [9,10,11,12,13]. However, this immune response is often inefficient, as cancer cells can evade immunological recognition and destruction by activating coinhibitory pathways through the molecular interactions of immune checkpoint proteins [14,15,16,17].

Programmed death 1 (PD-1) is a coinhibitory receptor on the T-cell surface and its primary biological function is to maintain peripheral tolerance by suppressing T-cell activation [9,18]. Upon binding to its ligands PD-L1 and PD-L2, the PD-1 receptor transmits inhibitory signals to T cells by recruiting the tyrosine phosphatase SHP2, thereby dephosphorylating proximal signaling elements [19,20,21]. While PD-1 is highly expressed on T cells of cancer patients, PD-L1 is often overexpressed in cancer cells, enabling cancer cells to evade immune surveillance by T cells. In addition, PD-L1 expression by cancer cells can directly induce the death of antigen-specific effector T cells expressing PD-1 [22]. Cytotoxic T lymphocyte-associated antigen 4 (CTLA-4) is a member of the CD28-B7 superfamily and negatively regulates T-cell activation. CTLA-4 functions to inhibit T-cell activity through competing with the costimulatory receptor CD28 for binding with their common ligands, B7-1 and B7-2, for which CTLA4 has significantly higher affinity than CD28 [23,24]. Through this competition for B7 ligands, CTLA-4 attenuates CD28-mediated costimulatory signaling, which is primarily transduced by PI3K and AKT [25,26,27].

**Figure 1:**
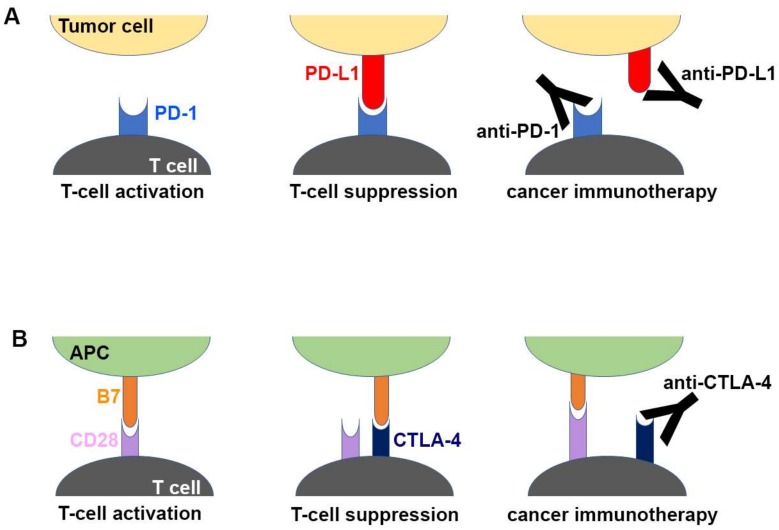
Schematic drawing of the molecular mechanism of checkpoint blockade by therapeutic antibodies for cancer immunotherapy. (**A**) T-cell activation is suppressed by the interaction between programmed death 1 (PD-1) on T cells and PD-L1 on tumor cells. Antibody drugs for cancer immunotherapy bind to PD-1 or PD-L1, blocking the PD-1/PD-L1 interaction. (**B**) T cells are activated by the interaction between B7 ligands of antigen-presenting cell (APC) and CD28 on T cells. Cytotoxic T lymphocyte-associated antigen 4 (CTLA-4) suppresses T-cell activation by competitive binding to B7. Therapeutic antibodies against CTLA-4 block the CTLA-4/B7 interaction.

Inhibition of the immune checkpoint blocks the inhibitory pathways of T-cell activation, thereby enabling tumor-reactive T cells to recognize tumor antigens and recover the antitumor immune response (Figure 1) [28,29]. Cancer immunotherapy using monoclonal antibodies against the immune checkpoint proteins, including PD-1 (and its ligand PD-L1) and CTLA-4, has demonstrated unprecedented therapeutic benefits and brought out a major breakthrough in oncology, helping to realize long-term durable responses in a subset of patients with multiple types of advanced cancers [30,31,32,33,34,35,36,37,38]. Since the FDA approval of ipilimumab, an anti-CTLA-4 antibody, in 2011 for the treatment of metastatic melanoma, six additional checkpoint-protein-blocking antibodies, all targeting the PD-1/PD-L1 axis, have been approved for treating a wide range of malignant cancers (Table 1). In addition, numerous combination therapies with these immune checkpoint inhibitors are under clinical trials to enhance therapeutic efficacy and response rate [16,39,40,41,42]. Although both CTLA-4 and PD-1 are negative regulators of T cells, each plays a nonredundant role in the coinhibitory mechanism of immune responses. While the interaction between CTLA4 and B7 ligands limits priming of naive T cells, the interaction between PD-1 and PD-L1 renders effector T cells to be exhausted in the tumor microenvironment, raising hopes for therapeutic synergy in the combination strategy [21,23]. In 2015, the combination of ipilimumab with nivolumab, an anti-PD-1 antibody, which showed an improved response rate compared with either monotherapy, was approved by the FDA for the treatment of melanoma [43]. Identification and validation of more reliable biomarkers would also improve the response rate by rational selection of cancer patients [44,45].

Structural studies of PD-1, PD-L1, CTLA-4, and their complexes have provided invaluable information for understanding the coinhibitory mechanism of T cells through the interaction of immune checkpoint proteins at the atomic level [46,47,48,49,50,51,52,53,54,55,56,57]. The extracellular domains of PD-1 and CTLA-4 are each composed of one Ig-like V-type domain, and their ligands contain two Ig-like domains each. The N-terminal domains of the ligands, including PD-L1, PD-L2, B7-1, and B7-2, are responsible for binding to PD-1 and CTLA-4. The crystal structures of human PD-1 in complex with human PD-L1, murine PD-1 in complex with murine PD-L2, and murine PD-1 in complex with human PD-L1 have established the structural foundations of the interactions of PD-1 with PD-L1 and PD-L2. The crystal structure of the CTLA-4 ectodomain revealed an unusual dimerization mode on the opposite side of its ligand binding site. The structure of CTLA-4 in complex with its ligands—B7-1 and B7-2—revealed the formation of a unique periodic arrangement between bivalent dimers of CTLA-4 and B7 ligands, providing insight into the formation of unusually stable signaling complexes for the negative regulation of T-cell immune responses within the immunological synapse.

Recently, the crystal structures of the therapeutic antibodies directly targeting the immune checkpoint molecules—PD-1, PD-L1, and CTLA-4—have been reported (Table 2) [58,59,60,61,62,63,64,65,66,67,68]. In this review, we primarily discuss the structural features and the interactions of these monoclonal antibodies related to their clinical cancer-treatment efficacies.

## 2. Structures of Anti-PD-1 Antibodies

In 2014, two antibody drugs against PD-1—pembrolizumab and nivolumab—were approved by the FDA for the treatment of melanoma and their indications have been expanded to a wide range of cancers, including non-small-cell lung cancer (NSCLC), renal cell carcinoma, Hodgkin lymphoma, head and neck squamous cell carcinoma, urothelial carcinoma, hepatocellular carcinoma, and gastric carcinoma, through additional clinical trials [39]. In 2018, an additional anti-PD-1 antibody, cemiplimab, was approved for patients with metastatic cutaneous squamous cell carcinoma [69]. All these anti-PD-1 antibodies are IgG4 antibodies for reducing any potential for the effect functions including antibody-dependent cellular cytotoxicity (ADCC) or complement-dependent cytotoxicity (CDC).

**Figure 2:**
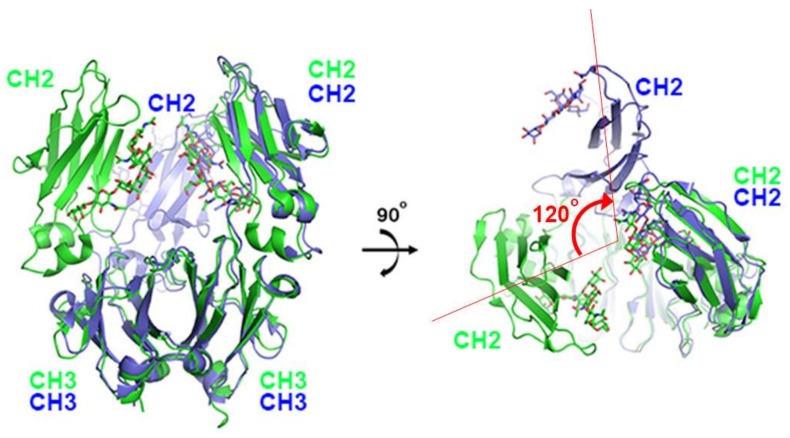
Structural comparison of pembrolizumab crystallizable fragment (Fc) (blue) in the full-length IgG4 with the isolated IgG4 Fc (green) on the surface of PD-1 in the complex structure. While the CH3 domains of both chains and the CH2 domains of one chain retain the same conformation, the CH2 domains of the other chain are ~120° apart. The glycans are shown in the stick model.

The first X-ray structure for an anti-PD-1 antibody was the full-length IgG4 structure of pembrolizumab [67]. This structure showed that one CH2 domain is rotated 120° relative to the position observed in the truncated crystallizable fragment (Fc), although the immunoglobulin fold was completely preserved (Figure 2). Both heavy chains of the Fc domain are glycosylated, but the glycan of the flipped CH2 domain faces the solvent. Pembrolizumab has a compact structure due to the presence of a short and rigid hinge region. The observed CH2 flipping of pembrolizumab can also be related to the constraints imposed by the short hinge region, as the usual CH2 conformation of the other Fc structure was incompatible with the relative positions of the Fc and Fab in pembrolizumab. Anti-PD-1 antibodies—pembrolizumab and nivolumab—are both IgG4 subclasses, which are by and large incapable of activating effect functions due to low affinity for complement component 1q (C1q) and Fc receptors. Although this structure provides significant insight into the structural feature of the full-length IgG4 subclass, the mechanism of action cannot be revealed due to the absence of PD-1 in the structure.

**Figure 3:**
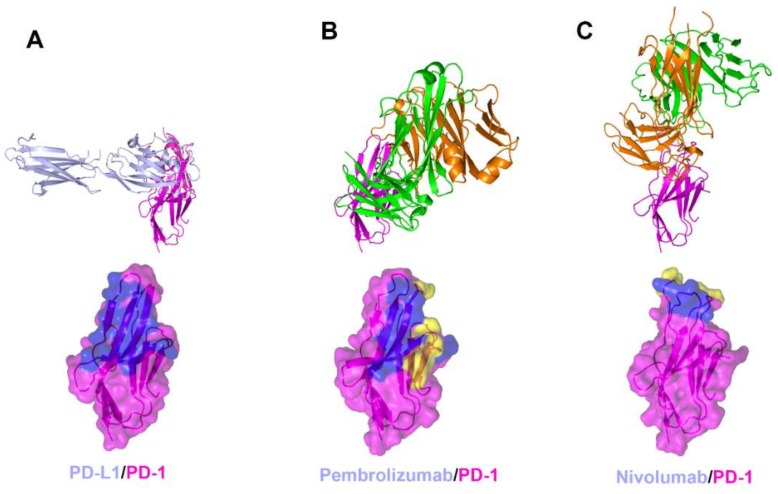
Crystal structures of PD-1 in complex with anti-PD-1 antibodies. (**A**) Structure of PD-1 (purple) in complex with PD-L1 (pale blue) and the PD-L1 binding site (blue) on the surface of PD-1. (**B**) Structure of PD-1 in complex with pembrolizumab and its epitope (yellow and blue) on the surface of PD-1. (**C**) Structure of PD-1 in complex with nivolumab and its epitope (yellow and blue) on the surface of PD-1. In (**A**–**C**), the ribbon models (top) and surface models (bottom) of PD-1 are displayed in the same orientation. The antibody heavy chain and light chain are colored green and orange, respectively, and the shared regions on the epitopes of the antibodies and the PD-L1 binding site are colored blue.

The interaction between PD-1 and the therapeutic antibodies were revealed by the crystal structures of PD-1 in complex with the antigen-binding fragment (Fab) or variable fragment (Fv) form of anti-PD-1 antibodies (Figure 3, Supplementary Table S1) [58,61,62,63]. The extracellular Ig-like V-type domain of PD-1 interacts with PD-L1 or PD-L2 via the residues of the C’CFG strands, which form a central β sheet. Crystal structures of PD-1 in complex with pembrolizumab and nivolumab have shown that there are significant overlaps between the epitopes of both antibodies and the PD-1 ligand binding site. The dissociation constants of pembrolizumab and nivolumab to PD-1 have been reported to be 27 pM and 1.45 nM, respectively, whereas the dissociation constant of the PD1/PD-L1 interaction was reported as 8.2 μM [48,49,61,62]. The total buried surface areas of the complexes of pembrolizumab and nivolumab are 2126 and 1487 Å^2^, respectively, compared with 1970 Å^2^ for the PD-1 in complex with PD-L1. A simple comparison of the values of the interface areas cannot explain this large discrepancy in the PD-1 binding affinity between anti-PD-1 antibodies and PD-L1. Previously, directed evolution on the surface of PD-1 by yeast surface display generated a PD-1 mutant that binds PD-L1 with a high affinity of 110 pM, implying that the low affinity of PD-1/PD-L1 interaction is probably due to the incomplete complementarity of the interaction [70]. The low affinity between PD-1 and PD-L1 would be more favorable for the transient interaction to timely modulate immune responses through reversible binding.

**Figure 4:**
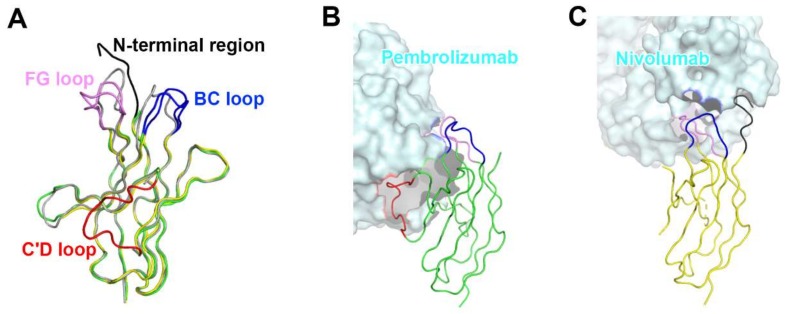
Conformational rearrangements of the loops of PD-1 induced by the binding of anti-PD-1 antibodies. (**A**) Superposition of PD-1 structures extracted from the complex structures of PD-1/PD-L1 (gray), PD-1/pembrolizumab (green), and PD-1/nivolumab (yellow). (**B**) Interactions of the loops of PD-1 with pembrolizumab. (**C**) Interactions of the loops of PD-1 with nivolumab. The *N*-terminal region of PD-1 in the PD-1/nivolumab is colored black. The BC, FG, and C’D loops in the PD-1/antibody complexes are colored blue, pink, and red, respectively. In (**B**, **C**), the PD-1 structures are displayed in the same orientation, and the antibodies in the complex structures are represented as cyan surfaces.

**Figure 5:**
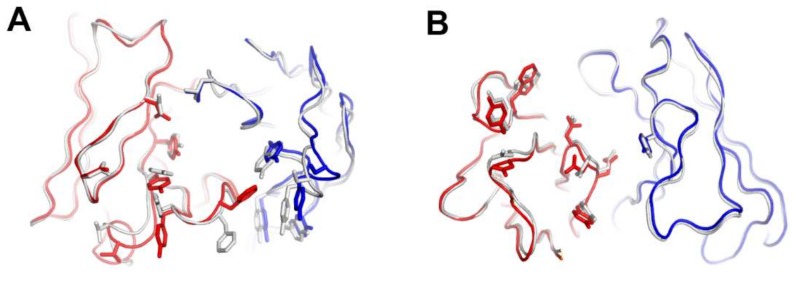
Structural comparison of the variable fragment (Fv) regions of anti-PD-1 antibodies before and after binding to PD-1. (**A**) Superposition of the Fv region of free pembrolizumab antigen-binding fragment (Fab) (PDB code 5dk3) onto that of pembrolizumab in complex with PD-1. (**B**) Superposition of the Fv region of free nivolumab Fab onto that of nivolumab in complex with PD-1. The heavy and light chains of the Fab fragments in the complexes are colored red and blue, respectively. The heavy and light chains in apo form are colored gray.

The interaction of pembrolizumab with PD-1 is primarily dependent on the C′D loop, which is highly flexible in the nuclear magnetic resonance (NMR) structure of free PD-1 and disordered in other PD-1 crystal structures due to a lack of interactions (Figure 4) [47,48,49,50,51,52]. This C′D loop intrudes into a groove formed by the paratope of pembrolizumab and most residues of the loop are involved in the interaction with pembrolizumab. In addition, pembrolizumab interacts with the C and C′ strands of PD-1, ensuring that it competes with the binding of PD-1 ligands [58,61,63]. The epitope of nivolumab is distinctly smaller than that of pembrolizumab and nivolumab does not interact with the C’D loop of PD-1 at all. Nivolumab binds PD-1 mainly through the *N*-terminal extension, which is located outside the Ig-like V-type domain of PD-1 and is not involved in PD-L recognition [58,62]. The *N*-terminal extension of PD-1 was not observed in the crystal structures of apo PD-1 or complexes with PD-L1, PD-L2, and pembrolizumab, implying its intrinsic flexibility. The binding site of the complementarity-determining regions (CDRs) on the light chain of nivolumab is located on the FG loop of PD-1, which overlaps the PD-1 binding site. In addition, the binding of pembrolizumab and nivolumab both induce conformational rearrangements in the flexible BC and FG loops of PD-1, which are not compatible with PD-L1 binding as they are also involved in the PD-L1 interaction in different conformations. These flexible loops within PD-1, including the *N*-terminal extension as well as the C’D, BC, and FG loops, are more likely to be recognized by antibody drugs and they can be promising epitopes for future therapeutic antibodies targeting PD-1. Interestingly, there is almost no overlap between the epitopes of pembrolizumab and nivolumab. Comparative binding analysis showed partial complementary binding of nivolumab to the pembrolizumab-PD-1 complex, and vice versa [62]. However, whether the combination of these two antibodies would elicit an improved therapeutic benefit is questionable given their high binding affinity to PD-1 at the picomolar range, compared to the micromolar affinity between PD-1 and PD-L1. The crystal structures of the free Fab fragments of pembrolizumab and nivolumab enabled the structural comparison of their Fv regions before and after binding to PD-1, showing little deviation in the conformation of the CDRs and minor adjustments in the side chains involved in the PD-1 interaction. This implies that the Fv regions of both antibodies maintain the CDR loops in productive conformation prior to binding to PD-1, partially contributing to the high binding affinities to PD-1 at a similar low picomolar range (Figure 5).

As pembrolizumab and nivolumab are IgG4, which is the preferred isotype for immunotherapy where recruitment of the effector functions such as ADCC and CDC is unwanted, their proposed mechanism of action is singular: blockade of the interaction between PD-1 and its ligands through outcompeting PD-L1 for binding to PD-1. Therefore, any differences in the clinical efficacy between nivolumab and pembrolizumab would not primarily originate from the differences in the specific interactions of their epitopes.

## 3. Structures of Anti-PD-L1 Antibodies

In 2016 and 2017, the FDA approved three anti-PD-L1 antibodies—atezolizumab, durvalumab, and avelumab—for the treatment of urothelial/bladder cancer, NSCLC, and Merkel cell cancer. While atezolizumab and durvalumab are IgG1 antibodies engineered to reduce the Fc-mediated effector functions, avelumab retains the potential to induce ADCC in its IgG1 form. Another therapeutic antibody targeting PD-L1, BMS-936559, is a fully human IgG4 antibody under clinical trials [71,72].

**Figure 6:**
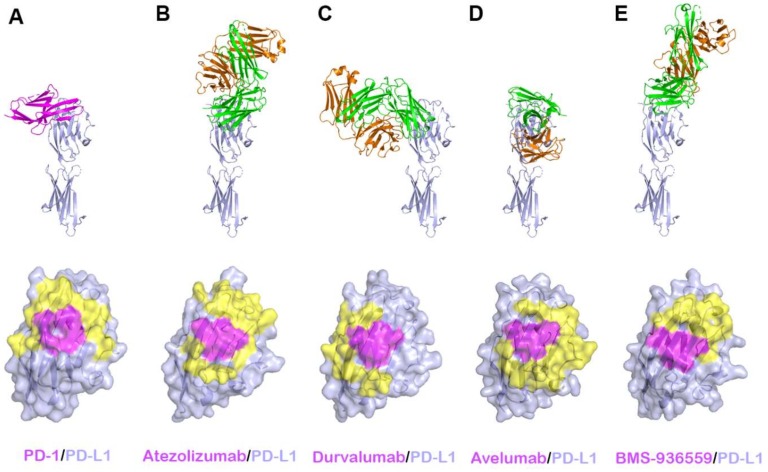
Crystal structures of PD-L1 in complex with anti-PD-L1 antibodies. (**A**) Structure of PD-L1 (pale blue) in complex with PD-1 (purple) and the PD-1 binding site (yellow and purple) on the surface of the IgV domain of PD-L1. (**B**) Structure of PD-L1 in complex with atezolizumab and its epitope on the surface of PD-L1. (**C**) Structure of PD-L1 in complex with durvalumab and its epitope on the surface of PD-L1. (**D**) Structure of PD-L1 in complex with avelumab and its epitope on the surface of PD-L1. (**E**) Structure of PD-L1 in complex with BMS-936559 and its epitope on the surface of PD-L1. In (**A**–**E**), the ribbon models (top) and surface models (bottom) of PD-L1 are displayed in the same orientation, and the antibody heavy and light chains are colored green and orange, respectively. The shared regions on the epitopes of the four antibodies and the PD-1 binding site are colored purple.

Recently, the crystal structures of PD-L1 in complex with all four of these antibodies have been determined, elucidating the precise epitopes and binding modes for the antigen–antibody interactions [58,64,65,66,68]. A comprehensive comparative analysis of the interactions of anti-PD-L1 antibodies with PD-L1 enables us to understand the blockade mechanism of these antibodies and provides a basis for the improvement of therapeutic antibodies against PD-L1 (Figure 6, Supplementary Table S2). The Fab fragments of atezolizumab and BMS-936559 bind to the upper side close to the *N*-terminus of PD-L1, heavily tilted toward the face containing the central CC’FG sheet, while durvalumab and avelumab bind rather perpendicularly to the face of PD-L1. The total buried surface areas of the complexes of atezolizumab, durvalumab, avelumab, and BMS-936559 are 2106, 1624, 1865, and 1349 Å^2^, respectively. The binding affinities of atezolizumab, durvalumab, and avelumab to PD-L1 have been reported to be 400, 42, and 667 pM, respectively [65,66,73]. Given that the buried surface area of PD-1/PD-L1 is 1970 Å^2^, the high affinities of these anti-PD-L1 antibodies should originate from their high complementary interactions with PD-L1. Although the antibodies bind PD-L1 in various binding orientations and with different epitopes from each other, they interact with the five hotspot residues (Y56, E58, R113, M115, and Y123) on the central CC’FG β sheet within PD-L1. These residues also play pivotal roles for the interaction between PD-1 and PD-L1, implying that the mechanism of these anti-PD-L1 antibodies involves outcompeting PD-1 for binding to PD-1 through the overlap of their epitopes within the central CC’FG sheet of PD-L1. In addition, the increased avidity from IgG bivalency could also contribute to the therapeutic efficacy of anti-PD-L1 antibodies due to the high expression level of PD-L1 in tumors. In addition to the CC’FG β sheet, the loop regions within PD-L1 also provide additional binding energy to stabilize the antigen–antibody complexes. Specifically, the BC, CC′, C′C″, and FG loops within PD-L1 make extensive interactions with atezolizumab, and the residues of the CC′ loop and N-terminal region are involved in the interaction with durvalumab. While the BC, C′C″, and FG loops of PD-L1 provide key interactions with BMS-936559, the CC′ loop makes a major interaction with avelumab through multiple hydrogen bonds. Unlike PD-1, within which the N-terminal extension, C′D, BC, and FG loops are very flexible and adopt distinct conformations with different binding partners; these loops in the complex structures adopt similar conformations as in the free PD-L1 structure, implying that PD-L1 is a relatively rigid molecule. Taken together, the BC, CC′, C′C″, and FG loops, as well as the central CC′FG β sheet, can be potentially recognized by therapeutic antibodies targeting PD-L1.

**Figure 7:**
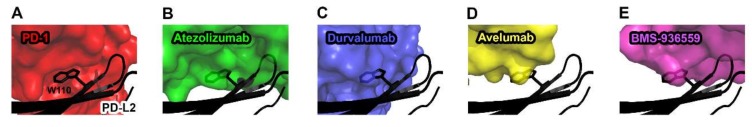
Structural basis for the lack of the binding of anti-PD-L1 antibodies to PD-L2. (**A**) The interaction of Trp110 of PD-L2 (black) with the surface of PD-1 (red) in the PD-1/PD-L2 complex (PDB code 3bov). (**B**) The partially transparent surface model of atezolizumab (green) when PD-L1 of PD-L1/atezolizumab is overlaid onto PD-L2. (**C**) The partially transparent surface model of durvalumab (blue) when PD-L1 of PD-L1/durvalumab is overlaid onto PD-L2. (**D**) The partially transparent surface model of avelumab (yellow) when PD-L1 of PD-L1/avelumab is overlaid onto PD-L2. (**E**) The partially transparent surface model of BMS-963559 (purple) when PD-L1 of PD-L1/BMS-963559 is overlaid onto PD-L2. In (**B**–**E**), Trp110 of PD-L2 (black) sterically collides with the surfaces of the antibodies.

While anti-PD-1 antibodies block the interaction of PD-1 with both PD-L1 and PD-L2, the anti-PD-L1 antibodies atezolizumab, durvalumab, avelumab, and BMS-936559 selectively inhibit the PD-1/PD-L1 interaction without disturbing the PD-1/PD-L2 interaction [73]. As PD-L2 plays a role in maintaining immune tolerance in the lung through interaction with PD-1, selective blockade of the PD-1/PD-L1 by the anti-PD-L1 antibodies is expected to avoid any toxicity associated with PD-L2 inhibition [74]. The crystal structures of PD-L1 in complex with these antibodies provide the basis of their selectivity to PD-L1 (Figure 7). In the crystal structure of the PD-1/PD-L2 complex, PD-L2 has similar binding modes to PD-1 as PD-L1 [51]. However, Trp110 of PD-L2, which is located within the G strand, plays a pivotal role in the interaction with PD-1, while the residue Ala121 of PD-L1, which corresponds to Trp110 of PD-L2, leads to a lower binding affinity of PD-L1 to PD-1. The complex structures of PD-L1 with the antibodies showed structural complementarity between Ala121 of PD-L1 and the paratopes of all these antibodies. The bulky size of Trp110 of PD-L2 should sterically collide with the anti-PD-L1 antibodies, thereby leading to selective inhibition of PD-L1 due to the failure in binding to PD-L2.

## 4. Structures of Anti-CTLA4 Antibodies

**Figure 8:**
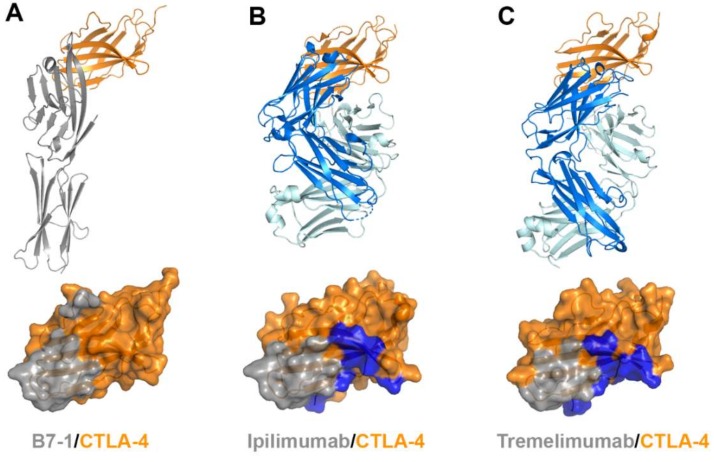
Crystal structures of CTLA-4 in complex with anti-CTLA-4 antibodies. (**A**) Structure of CTLA-4 (orange) in complex with B7-1 (gray) and the B7-1 binding site (gray) on the surface of CTLA-4. (**B**) Structure of CTLA-4 in complex with ipilimumab and its epitope (gray and blue) on the surface of CTLA-4. (**C**) Structure of CTLA-4 in complex with tremelimumab and its epitope (gray and blue) on the surface of CTLA-4. In (**A**–**C**), the ribbon models (top) and surface models (bottom) of CTLA-4 are displayed in the same orientation. The antibody heavy and light chains are colored blue and cyan, respectively, and the shared regions on the epitopes of the antibodies and the B7-1 binding site are colored gray.

Ipilimumab is the first-in-class immune checkpoint inhibitor targeting CTLA-4 and its FDA approval in 2011 initiated a new era in cancer immunotherapy. Tremelimumab, another anti-CTLA-4 antibody, is in phase III clinical trials. Notably, combination therapies of these anti-CTLA-4 antibodies with the antibodies against PD-1 or PD-L1 have shown significantly enhanced therapeutic efficacy in multiple clinical trials probably due to their different roles in T-cell inactivation. Recently, the crystal structures of CTLA-4 in complex with ipilimumab and tremelimumab have been reported, providing the molecular basis of CTLA-4 blockade by these antibodies (Figure 8, Supplementary Table S3) [58,59,60].

The extracellular domains CTLA-4 and CD28 are composed of a single immunoglobulin variable domain, sharing ~30% amino acid sequence identity [7]. They exhibit two β-sheet faces, the front face with the A′GFCC′ strands and the back face with the ABED strands [56]. The complex structures of CTLA-4 reveal that the front A′GFCC′ sheet provides the main residues for the interaction of CTLA-4 with its B7 ligands or the therapeutic antibodies [55,57]. The overlapping binding surface on CTLA-4 for B7, ipilimumab, and tremelimumab is predominantly located on the F and G strands of the front face. The recognition of the B7 ligands by CTLA-4 involves the FG loop of CTLA-4 with the sequence of MYPPPYY (residues 99–105). The crystal structures of the apo form of CTLA-4 and its complex with B7-1 or B7-2 show that the three consecutive proline residues within the FG loop adopt an unusual *cis-trans-cis* conformation and provide key interactions with the B7 ligands [53,54,55,56]. Indeed, mutation in the FG loop resulted in more than 90% loss of binding affinity to the B7 ligands [75]. In the complex structures of CTLA-4 with ipilimumab and tremelimumab, the FG loop is also involved in the interaction with the antibodies, but there is no substantial difference in its conformation from the structures of the apo form or B7-bound CTLA-4, suggesting that this loop region is rigid and ready for productive binding to its ligands or antibodies. The total buried surface areas of the complexes of ipilimumab and tremelimumab are 1880 and 1802 Å^2^, respectively, while 1255 Å^2^ for CTLA-4/B7-1 and 1212 Å^2^ for CTLA-4/B7-2. These differences in the total buried surface area upon binding CTLA-4 are consistent with the discrepancy of the binding affinities to CTLA-4 between the B7 ligand and the antibodies. The binding affinities of ipilimumab (K_d_ = 18 nM) and tremelimumab (K_d_ = 5.9 nM) are much higher than that of B7-1 (K_d_ = 420 nM) [59]. Therefore, ipilimumab and tremelimumab effectively compete with the B7 ligands for binding CTLA-4.

**Figure 9:**
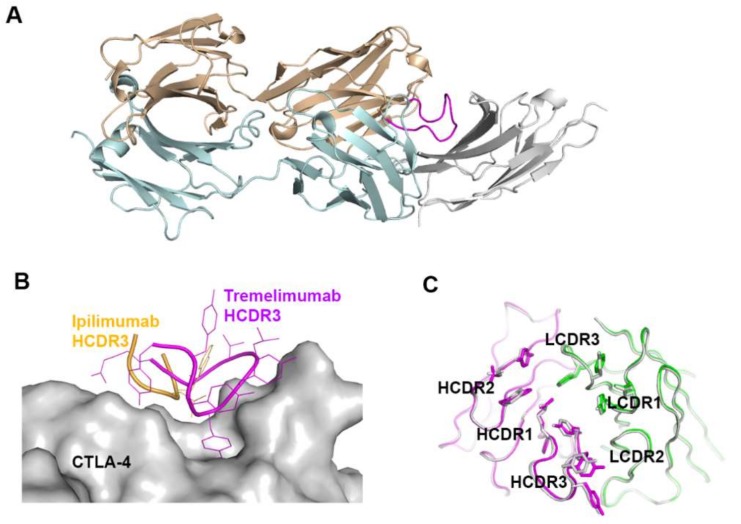
Exceptionally long HCDR3 loop of tremelimumab. (**A**) Complex structure of CTLA-4 (gray) and tremelimumab Fab. The HCDR2 of tremelimumab is colored purple. (**B**) Comparison of the interaction of HCDR3 between tremelimumab (purple) and ipilimumab (yellow) with CTLA-4 (gray). (**C**) Superposition of the Fv region of free tremelimumab Fab onto that of tremelimumab in complex with CTLA-4. The heavy and light chains of tremelimumab in the complex are colored purple and green, respectively. The heavy and light chains in free form are colored gray.

The comparison of the binding characteristics between ipilimumab and tremelimumab reveals remarkably similar binding orientations and epitopes of these two antibodies (Figure 8). However, the CDR3 loops on the heavy chain (HCDR3) are completely different from each other in their lengths and interactions with CTLA-4. The HCDR3 of tremelimumab (18 residues) is much longer than that of ipilimumab (10 residues) and contributes more to the interaction with CTLA-4 (Figure 9). Nine of the 10 residues within the overhang (residues 101–110) of tremelimumab HCDR3 are involved in the interaction with CTLA-4, tightly occupying the groove on the surface of the epitope. The structure of the apo form of tremelimumab Fab shows that the conformation of the HCDR3 is substantially identical to that of the complex structure with bound CTLA-4, implying that antibody context is critical for the “preformed” conformation of the long HCDR3 through interactions with other CDRs and framework regions of tremelimumab.

**Figure 10:**
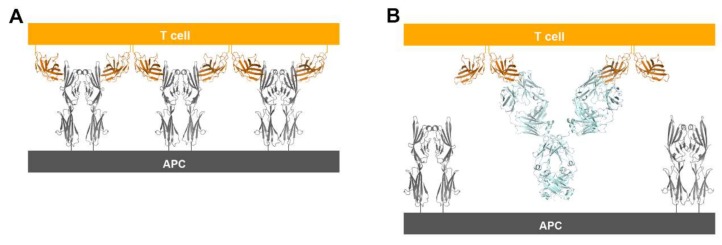
Suggestion of a model for prevention of the periodic arrangement of bivalent dimers of CTLA-4 and B7-1/2 by the binding of anti-CTLA-4 antibodies. (**A**) The alternating periodic arrangement of bivalent dimers of CTLA-4 and B7-1 at the interface between a T cell and APC. (**B**) Suggested model for the bivalent interaction of the anti-CTLA-4 antibodies with CTLA-4. An antibody binds two CTLA-4 molecules by bivalency of IgG.

CTLA-4 exists as a homodimer via an intermolecular disulfide bond [76]. In both structures of CTLA-4 in complex with ipilimumab and tremelimumab, CTLA-4 is presented as a homodimer identical to the previously reported structures of CTLA-4, implying that the binding by these antibodies does not affect the dimer formation. The crystal structures of CTLA-4 in complex with B7 ligands showed a unique periodic arrangement through the alternating interactions of bivalent CTLA-4 homodimers with bivalent B7 homodimers, providing an assembly model of CTLA-4 and B7 ligands within the immunological synapse between a T cell and an antigen-presenting cell (APC) [53,55]. This oligomeric array of the CTLA-4/B7 complex is supposed to promote coinhibitory signaling by clustering low-abundance CTLA-4 on the T-cell surface and decreasing the local concentration of CD28 through simple steric crowding. Given the similar binding modes of ipilimumab and tremelimumab, the modes of bivalent interaction of their IgG forms with CTLA-4 would be also similar (Figure 10). The dimension of the CTLA-4/antibody complex would lead to an intercellular distance, which is incompatible with the oligomeric arrangement of the CTLA-4/B7 complex, preventing or disrupting the assembly of the CTLA-4/B7 complex. These findings suggest that the therapeutic efficacy of these anti-CTLA-4 antibodies is a consequence of not only the simple antagonism of the interaction between CTLA-4 and B7 ligands but also the disruption of the unique assembly of the CTLA-4/B7 complex, which is an efficient arrangement for the coinhibitory signaling of CTLA-4.

**Figure 11:**
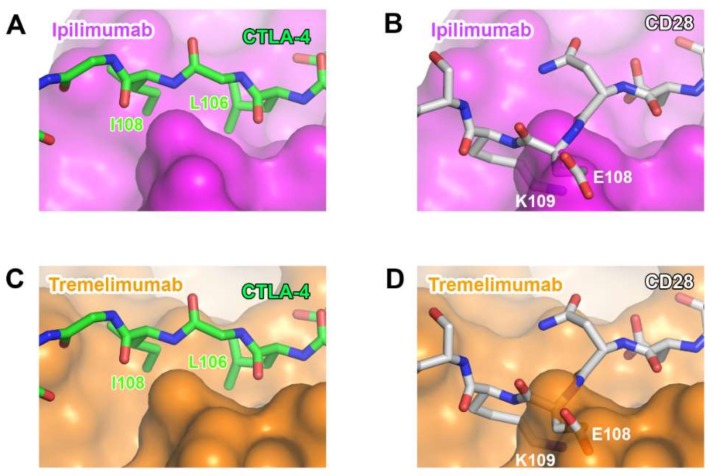
Structural basis for the lack of the binding of anti-CTLA-4 antibodies to CD28. (**A**) The interaction of the G strand of CTLA-4 (green) with the surface of ipilimumab (purple). (**B**) The partially transparent surface model of ipilimumab (purple) when CD28 (white) is overlaid onto CTLA-4 of CTLA-4/ipilimumab. (**C**) The interaction of the G strand of CTLA-4 (green) with the surface of tremelimumab (orange). (**D**) The partially transparent surface model of tremelimumab (orange) when CD28 (white) is overlaid onto CTLA-4 of CTLA-4/tremelimumab.

Although CTLA-4 and CD28 have contrasting functions in T-cell immunity, they share B7 ligands as their binding counterparts. As their overall structures are also similar to each other due to the sequence homology, the ability of anti-CTLA-4 antibodies to discriminate between CTLA-4 and CD28 is essential for their therapeutic efficacy. Although many residues of CTLA-4 involved in its interactions with ipilimumab and tremelimumab are conserved in CD28, there are critical differences within the G strand, which allows key interactions for the antibody binding. In CD28, a single residue Glu108 is inserted just before the G strand, thereby perturbing the interstrand interaction between the F and G strands and causing the protrusion of the G strand when compared with the CTLA-4 structure [77]. Superposition of CD28 onto the structures of CTLA-4 in complex with ipilimumab and tremelimumab shows that the insertion of Glu108 and the distortion of the G strand can cause a substantial steric collision with the surfaces of the antibodies, providing the structural basis for the CTLA-4 specificity exhibited by ipilimumab and tremelimumab (Figure 11).

Recent studies suggest that the therapeutic efficacy of anti-CTLA-4 antibodies is achieved through two mechanisms: blockade of the interaction between CTLA-4 and B7 ligands and depletion of intratumoral Treg cells through Fc-mediated effector functions including ADCC and CDC [59,60]. Ipilimumab is an IgG1 isotype with strong capability of ADCC/CDC, while tremelimumab is IgG2, which would less likely induce Fc-mediated effector functions [78]. However, tremelimumab also has shown antitumor activity in clinical trials of its mono- and combination therapies [79]. In the future, the accumulation of such structural studies with other anti-CTLA-4 antibodies can enhance the understanding of the mechanism related to the therapeutic efficacy of anti-CTLA-4 antibodies in cancer immunotherapy.

## 5. Conclusions

The Nobel Prize in Physiology or Medicine 2018 was awarded for the discovery of cancer therapy by the inhibition of CTLA-4 and PD-1. The successful development of immune checkpoint inhibitors targeting PD-1 (nivolumab, pembrolizumab, and cemiplimab), PD-L1 (atezolizumab, durvalumab, and avelumab), and CTLA-4 (ipilimumab) was based on the scientific research that identified the coinhibitory mechanism of T-cell activation by immune checkpoints. Recent structural studies on the therapeutic antibodies targeting PD-1, PD-L1, and CTLA-4 have improved our understanding of the molecular mechanisms underlying the antitumor activities of these antibodies, thereby providing invaluable information needed for the rational development of better immunotherapies of cancer. The epitopes and binding modes of these antibodies can be references for the development of other antibodies in the future. Given that the binding kinetics (i.e., k_on_, k_off_, and K_d_) of therapeutic antibodies is one of the most important determinants for the ultimate therapeutic function, these structures can aid in controlling the surface complementarity of the interface between antibodies and immune checkpoints, and thereby optimize the binding affinity for more selective and efficacious therapeutic properties through the rational design of affinity-modulated variants of the antibodies against PD-1, PD-L1, and CTLA-4. Moreover, a comprehensive comparison of the interactions of antibodies targeting immune checkpoint proteins revealed the epitope diversity, providing a better understanding of the molecular mechanism of the immune checkpoint inhibitors. Different mechanisms of action of therapeutic antibodies can lead to different clinical results. Therefore, precise understanding of the mechanisms of action of therapeutic antibodies through structural studies can provide a rationale for a better design of combination immunotherapies to achieve a synergistic antitumor effect. In addition, these complex structures can facilitate the design of small molecules targeting immune checkpoints by mimicking the diverse interactions of these therapeutic antibodies, providing an alternative treatment strategy to overcome the specific shortcomings of antibody-based drugs, such as the high manufacturing cost, limited half-life, immunogenicity, and poor diffusion into tumors.

## Figures and Tables

**Table 1:** FDA-approved immune checkpoint blocking antibodies.

Target	Antibody Drug	Trade Name	Tumor Type (FDA Approval Year)
PD-1	Nivolumab (IgG4)	Opdivo	Melanoma (2014)
			Non-small-cell lung cancer (2015)
			Hodgkin lymphoma (2016)
			Head and neck squamous cell carcinoma (2016)
			Urothelial carcinoma (2017)
			Hepatocellular carcinoma (2017)
	Pembrolizumab (IgG4)	Keytruda	Melanoma (2014)
			Non-small-cell lung cancer (2015)
			Head and neck squamous cell carcinoma (2016)
			Hodgkin lymphoma (2017)
			Urothelial carcinoma (2017)
			Gastic and gastroesophageal carcinoma (2017)
	Cemiplimab (IgG4)	Libtayo	Cutaneous squamous cell carcinoma (2018)
PD-L1	Atezolizumab (IgG1)	Tecentriq	Urothelial carcinoma (2016)
			Non-small-cell lung cancer (2016)
	Durvalumab (IgG1)	Imfinzi	Urothelial carcinoma (2017)
			Non-small-cell lung cancer (2018)
	Avelumab (IgG1)	Bavencio	Merkel cell carcinoma (2017)
			Urothelial carcinoma (2017)
CTLA-4	Ipilimumab (IgG1)	Yervoy	Melanoma (2011)

**Table 2:** Crystal structures of therapeutic antibodies against PD-1, PD-L1, and CTLA-4.

Target	Antibody	Structured Domains (Antibody/Target)	PDB ID	References
PD-1	Nivolumab	Fab fragment	5GGQ	[58]
		Fab fragment/Ig-like V-type extracellular	5GGR	[58]
		Fab fragment/Ig-like V-type extracellular	5WT9	[62]
	Pembrolizumab	Full length IgG4	5DK3	[67]
		Fab fragment/Ig-like V-type extracellular	5GGS	[58]
		Fab fragment/Ig-like V-type extracellular	5JXE	[61]
		Fv/Ig-like V-type extracellular	5B8C	[63]
PD-L1	Atezolizumab	Fab fragment/Ig-like V-type extracellular	5X8L	[64]
		Fab fragment/Ig-like V-type extracellular	5XXY	[68]
	Durvalumab	Fab fragment/Ig-like V-type extracellular	5X8M	[64]
		scFV/Ig-like V-type and Ig-like C2-type extracellular	5XJ4	[65]
	Avelumab	scFV/Ig-like V-type and Ig-like C2-type extracellular	5GRJ	[66]
	BMS-936559	Fab fragment/Ig-like V-type extracellular	5GGT	[58]
CTLA-4	Ipilimumab	Fab fragment/Ig-like V-type extracellular	5TRU	[60]
		scFv/Ig-like V-type extracellular	5XJ3	[59]
	Tremelimumab	Fab fragment	5GGU	[58]
		Fab fragment/Ig-like V-type extracellular	5GGV	[58]

## Supplementary Materials

The supplementary materials are available online.
