# Supplementary material for: Molecular Interactions of Antibody Drugs Targeting PD-1, PD-L1, and CTLA-4 in Immuno-Oncology
Source: Molecules. 2019 Mar 26;24(6):1190. doi: 10.3390/molecules24061190 (PMC6470598; doi:10.3390/molecules24061190)
Supplement: Supplementary file 1 [file molecules-24-01190-s001.pdf]

SUPPLEMENTARY INFORMATION

Molecular Interactions of Antibody Drugs Targeting PD-1, PD-L1, and CTLA-4 in Immuno-oncology

Hyun Tae Lee, Sang Hyung Lee and Yong-Seok Heo *

Department of Chemistry, Konkuk University, 120 Neungdong-ro, Gwangjin-gu, Seoul 05029, Korea
hst2649@naver.com (H.T.L.); dltkdgud92@naver.com (S.H.L.)
*Correspondence: ysheo@konkuk.ac.kr; Tel.: +82-2-450-3408; Fax: +82-2-3436-5382

Supplementary Table S1. Residues involved in the interactions between PD-1 and anti-PD-1.

PD-1	Pembrolizumab	Nivolumab
**S27**		$_H$Y53
**P28**		$_H$Y53, $_H$W52, $_H$G33
**D29**		$_H$N99, $_H$N31
**R30**		$_H$N31
**P31**		$_H$Y53
**S60**		$_H$N31, $_H$T28
**E61**		$_H$T28, $_H$G26
**S62**	$_L$Y57	
**V64**	$_H$M105	
**Y68**	$_H$Y101, $_H$R102	
**Q75**	$_H$T30	
**T76**	$_H$Y101	
**D77**	$_H$T30, $_H$S54	
**K78**	$_H$N52, $_H$Y33, $_H$D108	
**A81**	$_H$M105	
**F82**	$_H$F103, $_L$S32	
**P83**	$_H$F103, $_L$Y34	
**E84**	$_L$Y36	
**D85**	$_H$R99	
**R86**	$_L$S95, $_L$L100	
**S87**	$_H$R99, $_H$N59, $_H$Y35, $_L$L100	
**Q88**	$_H$Y35, $_H$Y33	
**P89**	$_H$Y35	
**G90**	$_H$T58, $_H$Y35, $_H$T58	
**L128**	$_H$Y34, $_H$F103, $_H$M105, $_L$Y57	$_H$G26
**A129**	$_H$M105	$_H$I27, $_H$Y102, $_L$T56
**P130**		$_L$A55, $_L$L46
**K131**	$_H$M105	$_H$N99, $_L$S91, $_L$Y49
**A132**	$_H$R102	$_L$Y49, $_H$I27
**Q133**		$_H$T28
**I134**	$_H$R102	

The antibody residues, which make hydrogen bonding or salt bridge with PD-1, are colored red.

Supplementary Table S2. Residues involved in the interactions between PD-L1 and anti-PD-L1.

PD-L1	Atezolizumab	Durvalumab	Avelumab	BMS-936559
**A18**	$_{H}$P102			
**T20**		$_{L}$S94, $_{L}$L95		
**V23**		$_{L}$S30		
**D26**		$_{L}$R28		
**E45**	$_{L}$S30			
**D49**	$_{L}$Y93			$_{H}$G105, $_{L}$Y32, $_{H}$S104
**A51**	$_{L}$Y93			$_{H}$S106, $_{L}$S92
**A52**	$_{L}$L92, $_{L}$Y93			$_{H}$P107, $_{H}$K99
**I54**	$_{H}$W33, $_{H}$W50		$_{H}$S54, $_{H}$G55	$_{H}$I52, $_{H}$F108
**Y56**	$_{H}$W33, $_{H}$W50, $_{H}$S57	$_{H}$S56	$_{H}$Y52, $_{H}$P53, $_{H}$S54, $_{H}$I57	$_{H}$H59, $_{H}$F55
**E58**	$_{H}$S52, $_{H}$S57	$_{H}$K52	$_{H}$I33, $_{H}$Y52, $_{H}$V104	$_{H}$K57
**M59**			$_{L}$Y34	
**E60**	$_{H}$Y54, $_{H}$T74	$_{H}$W102	$_{L}$Y32, $_{L}$Y34, $_{L}$Y93	
**D61**	$_{H}$G55, $_{H}$T74	$_{H}$W102	$_{L}$R99, $_{H}$V104, $_{H}$T105, $_{H}$T106	
**N63**	$_{H}$S57		$_{H}$G102, $_{H}$T103	
**Q66**	$_{H}$T58		$_{H}$S31, $_{H}$P53	$_{H}$H59
**V68**	$_{H}$W50, $_{H}$Y59			$_{H}$H59, $_{L}$W94
**H69**	$_{H}$Y59, $_{L}$H94		$_{H}$S54	$_{H}$S106, $_{L}$N93
**D73**			$_{H}$S31	
**K75**			$_{H}$F27, $_{H}$728, $_{H}$S31	
**V76**			$_{H}$G102	
**V111**	$_{H}$Y54	$_{L}$Y33, $_{H}$F103, $_{H}$E105		
**R113**	$_{H}$D31	$_{H}$E57	$_{H}$Y52, $_{H}$F53, $_{L}$S97	$_{H}$F55
**M115**	$_{H}$W33, $_{H}$W50, $_{H}$W101	$_{H}$Y59	$_{H}$Y52, $_{H}$I57	$_{H}$F55
**S117**	$_{H}$W33		$_{H}$G55, $_{H}$I57	$_{H}$I52
**Y118**	$_{L}$T31, $_{L}$L92			
**G119**	$_{L}$A32, $_{L}$Y91, $_{H}$R99			$_{H}$Y32
**G120**				$_{H}$Y32, $_{H}$T31
**A121**	$_{H}$W33, $_{H}$W101	$_{H}$Y59	$_{H}$I57	$_{H}$T31
**D122**		$_{L}$L95		$_{H}$I54
**Y123**	$_{H}$W101, $_{H}$D31	$_{L}$L95, $_{L}$W97, $_{H}$E99, $_{H}$F103	$_{H}$F53	$_{H}$I54
**K124**		$_{L}$S94		
**R125**	$_{H}$S30, $_{H}$D31	$_{L}$S31, $_{L}$Y92, $_{L}$G93, $_{H}$F103	$_{L}$S95, $_{L}$S97	
**T127**		$_{H}$W33		

The antibody residues, which make hydrogen bonding or salt bridge with PD-L1, are colored red.

Supplementary Table S3. Residues involved in the interactions between CTLA-4 and anti-CTLA-4.

CTLA-4	Ipilimumab	Tremelimumab
**K1**	$_L$Q27	$_L$N30, $_L$S28
**A2**		$_L$Q27, $_L$Y92,
**M3**	$_L$Y33	$_L$N30
**E33**	$_H$N57	$_H$N57
**R35**	$_H$Y53	$_H$Y53
**L39**	$_H$W101, $_H$L102	$_H$A103
**Q41**		$_H$Y107, $_H$Y105
**S44**	$_L$Y50	$_H$L105
**V46**	$_H$W101, $_H$L102	$_H$T104
**E48**	$_H$W101, $_H$S31	$_H$Y53
**L91**		$_H$Y107, $_H$T104
**I93**		$_H$Y107, $_H$G102
**K95**	$_H$W101, $_H$Y53	$_H$G102
**E97**	$_H$T33, $_H$Y53	$_H$R105, $_H$Y59
**M99**	$_H$S52, $_H$Y53, $_H$N57, $_H$Y59	$_H$Y59
**Y100**	$_H$N57, $_H$Y59	
**P101**	$_H$Y59	
**P102**	$_H$Y59	$_H$Y59
**P103**	$_L$S95	$_L$T94
**Y104**	$_H$F50, $_H$Y59, $_L$S94, $_L$S95, $_L$W97	$_H$Y59
**Y105**	$_L$G93, $_L$S94	$_L$Y92
**L106**	$_H$L102, $_H$G103, $_L$Y33, $_L$Y92, $_L$G93	$_H$Y106, $_H$Y110, $_H$R105
**I108**	$_H$W101, $_H$L102, $_L$Y33	$_H$Y110
**N110**		$_H$Y107, $_H$Y108, $_H$Y110

The antibody residues, which make hydrogen bonding or salt bridge with CTLA-4, are colored red.
